# FamPipe: An Automatic Analysis Pipeline for Analyzing Sequencing Data in Families for Disease Studies

**DOI:** 10.1371/journal.pcbi.1004980

**Published:** 2016-06-06

**Authors:** Ren-Hua Chung, Wei-Yun Tsai, Chen-Yu Kang, Po-Ju Yao, Hui-Ju Tsai, Chia-Hsiang Chen

**Affiliations:** 1 Division of Biostatistics and Bioinformatics, Institute of Population Health Sciences, National Health Research Institutes, Zhunan, Miaoli County, Taiwan; 2 Department of Public Health, China Medical University, Taichung, Taiwan; 3 Department of Pediatrics, Feinberg School of Medicine, Northwestern University, Chicago, Illinois, United States of America; 4 Department of Psychiatry, Chang Gung Memorial Hospital-Linkou, Gueishan, Taoyuan, Taiwan; 5 Department and Graduate Institute of Biomedical Sciences, Chang Gung University, Taoyuan, Taiwan; University of Canterbury, NEW ZEALAND

## Abstract

In disease studies, family-based designs have become an attractive approach to analyzing next-generation sequencing (NGS) data for the identification of rare mutations enriched in families. Substantial research effort has been devoted to developing pipelines for automating sequence alignment, variant calling, and annotation. However, fewer pipelines have been designed specifically for disease studies. Most of the current analysis pipelines for family-based disease studies using NGS data focus on a specific function, such as identifying variants with Mendelian inheritance or identifying shared chromosomal regions among affected family members. Consequently, some other useful family-based analysis tools, such as imputation, linkage, and association tools, have yet to be integrated and automated. We developed FamPipe, a comprehensive analysis pipeline, which includes several family-specific analysis modules, including the identification of shared chromosomal regions among affected family members, prioritizing variants assuming a disease model, imputation of untyped variants, and linkage and association tests. We used simulation studies to compare properties of some modules implemented in FamPipe, and based on the results, we provided suggestions for the selection of modules to achieve an optimal analysis strategy. The pipeline is under the GNU GPL License and can be downloaded for free at http://fampipe.sourceforge.net.

This is a *PLOS Computational Biology* Software article.

## Introduction

Next-generation sequencing (NGS) is now a popular technique for identifying novel rare variants that are potentially associated with diseases. The analysis of NGS data often requires the integration of various resources; hence, many analysis pipelines have been developed to facilitate this process. Substantial research effort has thus far been devoted to developing pipelines or workflows for automating sequence alignment, variant calling, and annotation. For example, 25 workflows and pipelines that served these purposes were identified by Pabinger et al. [1]. However, fewer pipelines have been designed specifically for disease studies. Those that exist include variant tools [2], which implement several popular statistical association tests, and VAAST 2.0 [3], which is based on an extended composite likelihood ratio test to prioritize variants.

Family-based studies are increasingly being conducted to identify rare disease susceptibility variants because a sufficient number of rare alleles that co-segregated with the disease can be observed in pedigrees [4]. Thus, several tools or pipelines have been developed for analyzing family-based NGS data. For Mendelian disorders, disease variants can be identified on the basis of the Mendelian inheritance rules (e.g., autosomal dominant or recessive or compound heterozygosity). Tools such as VAR-MD [5], FamAnn [6], and VariantDB [7] were designed to identify variants with Mendelian inheritance models. These tools, however, do not consider sequencing errors that can result in violations of the Mendelian inheritance rules for the disease variants. MendelScan [8] implements the segregation scores that can account for sequencing errors for prioritizing variants. On the other hand, the Shared Genomic Segment (SGS) method aims to identify haplotypes that are shared identical-by-descent among affected members within a family [9–11] and the method has been demonstrated to be powerful for finding rare disease variants [12]. Identity-by-descent (IBD) statistics for the SGS analysis can be calculated using tools such as Merlin [13] and MORGAN [14]. As generating input files of Merlin and MORGAN can become complicated, several tools were developed to assist with file preparation for the analyses with the two programs [15–17]. The Merlin output files can be further adopted by Olorin [18] for the SGS analysis. The main features in Olorin include the visualization of pedigree structures, identification of shared haplotypes among affected family members, and variant filtering in the sharing region based on the variant annotation information provided by the user. RVsharing calculates the exact probabilities of sharing by multiple affected relatives at variants under the null of no linkage and no association [19]. A test strategy based on the potential p-value, which is the highest exact probability from the probabilities for all families, is used to evaluate the significance of the exact probabilities.

In addition, linkage analysis provides statistical evidence supporting the roles of variants in diseases and can become a powerful approach for the analysis of sequencing data [20]. Some tools such as Merlin can perform exact computation for linkage analysis based on the Lander-Green algorithm [21] but are restricted to the use of small pedigrees. Hence, large pedigrees need to be split for the analysis [16]. Some other tools such as MORGAN use a Markov chain-Monte Carlo (MCMC)-based method that can accommodate large pedigrees and therefore do not require pedigree splitting [22].

Furthermore, tools for family-based association tests are available. Hu et al. [23] proposed pedigree-VAAST (pVAAST), which uses a composite likelihood ratio test incorporating linkage signal in families, external controls, and functional predictions of variants to identify variants with statistically significant associations with the disease. The application of pVAAST, however, is restricted by the test assumption that the external controls are from the same population as that of the family members and that these samples were sequenced on the same platform to maintain a correct type I error rate, as well as by the test requirement for a large set of external controls to achieve sufficient power (e.g., 1,000 external controls were generated in the simulation studies conducted by Hu et al. [23]). The weighted-sum statistic [24] also provides statistical test for genes associated with Mendelian disorders. The test also requires a large number of controls to achieve statistical power. Instead of using external controls, tools such as OVPDT [25], which accounts for both common and rare variants with different directions of effects on disease, and FBAT [26], which implements the weighted-sum approach [27], are available for family-based association analysis when the sample size is large. A review of several other family-based association tools can be found in Lee et al. [28].

Finally, imputation of untyped variants based on a subset of sequenced family members and a larger set of family members with SNP array data (e.g., data from genome-wide association studies (GWAS)) provides a cost-effective approach to increasing sample sizes [29]. Combining some of the aforementioned functions can form a powerful family-based analysis. For example, segregation scores can be used to rank variants in regions identified by the SGS analysis when searching for variants responsible for Mendelian disorders [8]. Moreover, if only a subset of family members were sequenced while a larger set of family members were genotyped with SNP arrays, family-based association tests using imputed genotypes can significantly increase the power compared with tests that use only the observed data [30]. However, one major challenge faced by researchers who are conducting family-based NGS data analyses is that without an automatic pipeline that integrates these functions, many tedious and inefficient steps need to be performed with in-house developed scripts. For example, genetic positions from resources such as Rutgers genetic map [31] and external population allele frequencies from resources such the 1000 Genomes Project [32] are required for Merlin and MORGAN. Scripts are also required to transform the output files from an imputation program to the input files for an association analysis tool.

To address the challenge faced by family-based NGS analysis for disease studies, we developed a pipeline, FamPipe, which can be applied to the analysis of Mendelian disorders or complex diseases. In particular, Merlin and MORGAN were integrated into FamPipe to calculate the IBD statistics or linkage LOD scores to identify linkage regions. For identifying variants responsible for Mendelian disorders, three methods were implemented in the disease model identification (DMI) module in FamPipe including the segregation scores [8], which can be used for identifying family-specific mutations at disease variants, the weighted-sum statistic [24], which is ideal for identifying mutations in multiple disease variants within a gene, and the filtering rules for compound heterozygosity [33]. For complex disease studies, family-based association tests can be performed in the linkage regions or across the whole genome. Furthermore, two family-based imputation tools, Merlin [34] and GIGI [29], are integrated into FamPipe for imputation analysis when the data consist of both sequencing and SNP array data.

## Design and Implementation

### FamPipe Modules

#### Allele Frequency Estimation (AFE) module

Population allele frequencies are required to determine minor alleles for disease model identification, to calculate the IBD and linkage likelihoods, and to infer haplotype frequencies in imputation analyses. Using the sample allele frequencies calculated from a few families as the estimates of the population allele frequencies may bias the statistical inference because minor alleles can be enriched in a family. For example, a rare mutation responsible for a recessive Mendelian disorder can be prevalent in families with the disease. Thus, we compiled several external allele frequency files using data from the 1000 Genomes Project [32] for different populations, including data from African, Admixed American, East Asian, European, and South Asian populations. Moreover, some variants have mutant alleles observed only in family samples but not in the external populations. Specifying the mutant allele frequencies as 0 for such variants can cause problems for statistical inference in tools such as Merlin. Therefore, a weighted allele frequency is estimated by considering the population and sample allele frequencies for each variant as follows:

$$f_{w}=\frac{nf_{s}+mf_{e}}{n+m}$$

where *f_s_* and *f_e_* are the allele frequencies for the allele in the sample and external frequency file, respectively, and *n* and *m* are the total allele counts in the sample and external populations. If an external frequency file is not specified, *f_w_* is equal to *f_s_*.

#### DMI module

Three strategies were implemented in the DMI module for identifying the variants responsible for Mendelian diseases. The first strategy aims to identify a variant with family-specific mutations inherited from a common ancestor associated with the disease. The goal of the second strategy is to identify a gene harboring several mutations at different disease causal variants in several families or unrelated affected individuals. Finally, the third strategy aims to identify compound heterozygosity for rare recessive diseases.

For the first strategy, the two segregation scores previously described in Koboldt et al. [8] are calculated for each variant assuming autosomal dominant and recessive models. The scores were designed for rare Mendelian disorders and allowed for genotyping errors so that genotypes violating the Mendelian rules still received some weight. Define *D* and *d* as minor and major alleles at a variant, respectively, based on the weighted allele frequencies from the AFE Module. Allele *D* is assumed as the rare disease allele. Under a dominant model, affected individuals with *DD* and *dd* are scored as 0.8 and 0.5, respectively, whereas unaffected individuals with *Dd* and *DD* are scored as 0.1 and 0.01, respectively. As described in Koboldt et al. [8], the scores reflected approximately 50% sensitivity, 20% miscall rate (heterozygous variants called homozygous), and 10% false positive rate. Under a recessive model, affected individuals with *Dd* and *dd* are scored as 0.5 and 0.1, respectively, whereas unaffected individuals with *DD* are scored as 0.1. Individuals with other genotypes are scored as 1. The segregation score for a variant assuming a certain disease model is the multiplication of scores at the variant for all individuals. The scoring parameters are the default parameters in the software MendelScan implementing the models in Koboldt et al. [8]. The parameter values can be changed by the user in FamPipe. In our simulation studies, the default parameter values were used.

For the second strategy, the weighted-sum statistic [24] and its p-value are calculated for each gene. The method has been shown to be powerful for identifying genes responsible for Mendelian diseases such as the Miller Syndrome, Freeman-Sheldon Syndrome, and Kabuki Syndrome using simulated sequencing data in a few affected individuals. Finally, the filtering rules for compound heterozygosity [33] were implemented in FamPipe, while some exceptions in the rules were allowed in FamPipe to accommodate different pedigree structures and genotyping errors. The first rule states that a variant has to be heterozygous in all affected individuals. The second rule states that a variant should not be homozygous disease allele in any of the unaffected individuals. The third rule states that only one of the parents can be heterozygous when their affected child is heterozygous. The first three rules are used at the variant level. At the gene level, the fourth rule states that a gene must have two or more variants following rules 1, 2, and 3. The fifth rule states that there must be at least one variant following rules 1, 2, and 3 transmitted from one parent and at least one variant following rules 1, 2, and 3 transmitted from the other parent. To allow for genotyping errors, we made relaxation on rules 1, 2, and 3 that only a certain proportion (e.g., 95%) of the affected individuals or children need to follow the rules. If a parent is affected, the fifth rule is not applicable as the disease alleles in an affected child will always have been transmitted from the affected parent. Therefore, we made an exception that the fifth rule can be excluded depending on the pedigree structures.

#### Update map module

Because genetic positions based on Haldane’s map function are required for Merlin, MORGAN and GIGI, the genetic position for each variant is updated in this Module based on the sex averaged Haldane’s position in Rutgers Map v.3a [31]. Genetic positions for variants not on Rutgers Map were linearly interpolated based on their physical distances.

#### Pedigree split module

Analyses of large extended pedigrees are restricted by the size of the computer memory in Merlin; therefore, these pedigrees are split into sub-pedigrees for the analyses in Merlin. A Pedigree Split Module that uses PedCut [35] was implemented to split a large pedigree into sub-pedigrees. A user-specified bit size for PedCut, calculated as twice the number of non-founders minus the number of founders, determines the number of family members in each sub-pedigree.

#### IBD module

Several studies have performed the SGS analysis for sequencing data using the IBD sharing statistics as one of the filters to identify chromosomal regions that are excessively shared among affected members within families [36–39]. This module calculates the proportion of pairs of affected familial members who share a chromosomal region in all pairs of affected family members. For every grid of the chromosomal region, probabilities of IBD states between every pair of relatives are estimated using Merlin. The grid size (e.g., 1 cM) is determined by the user. A pair of affected blood relatives with P(IBD≠0) for a region greater than a user-specified threshold (e.g., 0.5) is defined as an IBD pair for the region. Parent-offspring pairs are not considered as they always share one allele IBD. The proportion of IBD pairs in all pairs of affected blood relatives (excluding parent-offspring pairs), which is referred to as the IBD sharing statistic, is calculated for each variant. Regions with IBD sharing statistics greater than a user-specified threshold are defined as IBD regions.

#### Linkage module

In the Linkage Module, linkage LOD scores and p-values from one of the linkage tests provided by Merlin are calculated for every grid of the chromosomal region. As Merlin is restricted for the analysis of smaller pedigrees, for larger pedigrees, one of the linkage functions provided by MORGAN can be performed in FamPipe. The linkage functions in MORGAN include several IBD-based tests [40,41] and the estimation of location LOD scores [42,43]. Both Merlin and MORGAN assume that variants are independent for the linkage analysis. Therefore, we followed the criteria in PBAP [17], a suite of programs used to prepare files for pedigree-based analysis, to generate an informative and independent set of variants. Variants with minor allele frequencies (estimated from the AFE module) > 0.2 are selected. Then PLINK is used to perform linkage disequilibrium (LD) based pruning, using a variance inflation factor (VIF) value of 1. As suggested by the PLINK user manual, a VIF of 1 implies that the variants after pruning are completely independent. Moreover, if the genetic distance between a variant and the next variant is less than 0.5 cM, the next variant is removed. Regions with linkage LOD scores greater than a user-specified threshold or with IBD test p-values less than a user-specified threshold are defined as linkage regions.

#### Association module

If the sample size is large, conducting an association test is a powerful approach to identifying variants associated with the disease. Gene-based association tests based on OVPDT and FBAT are included in the Association Module. OVPDT considers the joint effects of both common and rare variants, as well as the direction of the effects of variants in a gene in nuclear families. In contrast, FBAT can analyze large pedigrees and uses a weighted-sum approach for rare variants in a gene, with the assumption that the rare variants have the same direction of effects on the disease. OVPDT and FBAT can be used as complementary tests for association analysis.

#### Imputation module

Two family-based imputation algorithms implemented in Merlin and GIGI were included in the Imputation Module. The imputation algorithms are useful for increasing the number of sequenced individuals when some pedigree members have been genotyped with only a sparse set of variants, such as the SNP array data, and when a subset of family members have been sequenced with a dense set of variants. Untyped variants in individuals with a sparse set of variants are imputed based on the dense set of variants and the IBD information inferred from the sparse data set. Merlin has been demonstrated to be useful for imputation in nuclear and three-generation families [34]. Moreover, Merlin can also handle other types of pedigrees as long as the bit size is not large (generally less than 20). By contrast, pedigree size is not limited in GIGI so that it can be used to impute large pedigrees.

GIGI requires a sparse set and a dense set of variants for imputation. Therefore, FamPipe expects one file that contains the sparse set of variants (e.g., the SNP array data) and another file that contains the dense set of variants (i.e., the NGS data). GIGI first uses MORGAN to infer inheritance vectors (IVs) based on the sparse set of variants. FamPipe therefore automatically generates input files for MORGAN. As MORGAN assumes that variants are independent for the inference of IVs, we also followed the criteria in PBAP to generate a set of informative and independent variants for MORGAN. MORGAN has several parameters for inferring the IVs. The recommended values in PBAP are also used by FamPipe. For example, the maximum number of meiosis for exact computation is set at 12, the L-sampler probability is set at 0.2, the number of Monte Carlo iterations is set at 100,000, and the burn-in iterations is set at 100. The threshold-based calling, which calls genotypes or alleles with probabilities greater than the specified thresholds, in GIGI is used in FamPipe. The default thresholds (i.e., t_1_ = 0.8 and t_2_ = 0.9) set by GIGI are used in FamPipe. Moreover, GIGI also generates an imputed genotype probability file, which has the genotype probabilities for each variant. These probabilities can be subsequently used in association analysis tools that accept such a format.

### Flowchart

**Fig 1:**
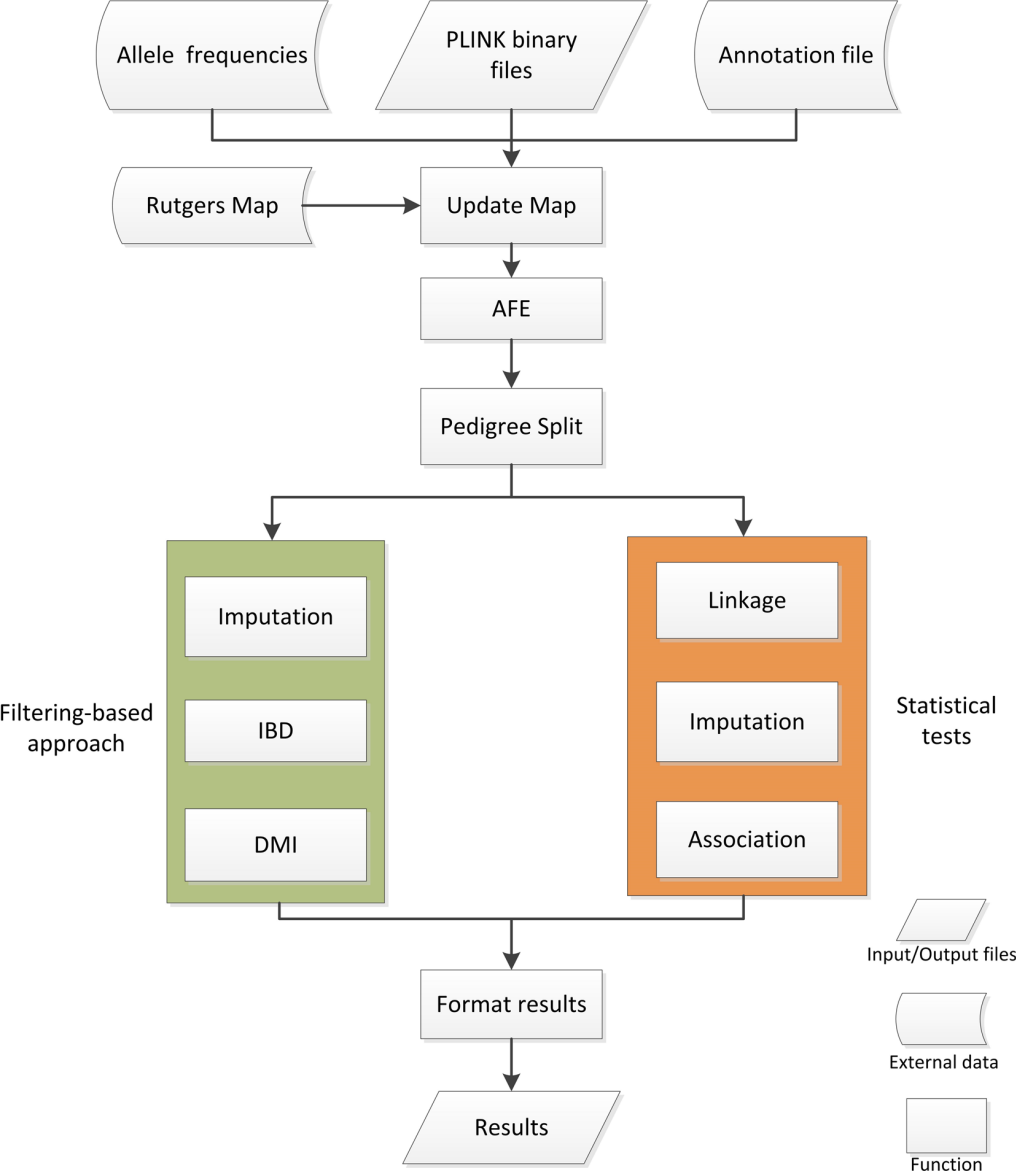
Flowchart of FamPipe.

Fig 1 shows the flowchart for FamPipe. FamPipe expects a set of binary files in the PLINK [44] format, which contain the variant calls, family structure, and variant information. The files may contain genotypes generated based on both SNP arrays and NGS. Optional files with information such as population allele frequencies and annotations are also accepted. The pipeline first runs the Update Map Module and sequentially runs the AFE Module. The Pedigree Split Module is executed if the dataset contains large pedigrees and Merlin will be performed for later analyses. If the sample contains only a few pedigrees and contains both SNP array and NGS data, the user can decide whether to perform the Imputation Module across the genome. Moreover, the filtering-based strategy employing modules such as the IBD and DMI Modules can be performed. If the sample size is large, statistical tests can be performed using the Linkage and Association Modules. Imputation is recommended to be performed in the previously identified linkage regions in this scenario because of the computational complexity of the imputation algorithms [29]. Association tests can be performed in linkage regions identified by the Linkage Module [45] or across the genome. Association tests can also be performed based on the imputed genotypes. Note that each module can be optionally executed to fulfill the analysis goal of the user. Finally, a results file, which contains the annotation information and statistics from each module being executed, is generated. A user-friendly web-based interface has been created for FamPipe (http://fampipe.sourceforge.net/generateCommand.html), and this can be used to easily generate a command line for running FamPipe in UNIX. If the input file contains multiple chromosomes, threads will be automatically executed to analyze the chromosomes in parallel in order to improve the analysis efficiency.

## Results

### Simulation Studies

The performance of using IBD-sharing statistics or linkage LOD scores to identify rare variants associated with Mendelian diseases has been evaluated in the literature [12,46], and detailed discussions and guidelines for applying the two approaches to sequencing data can also be found in the literature [4,20]. On the other hand, it is unclear how the three approaches implemented in the DMI module compare for Mendelian disease analysis using sequencing data. Therefore, we used simulations to evaluate the sensitivity and specificity of the three strategies implemented in the DMI module. Moreover, we also used simulations to evaluate the performance of the two family-based imputation tools (Merlin and GIGI) included in FamPipe. Details of the simulation study designs can be found in the S1 Text.

**Fig 2:**
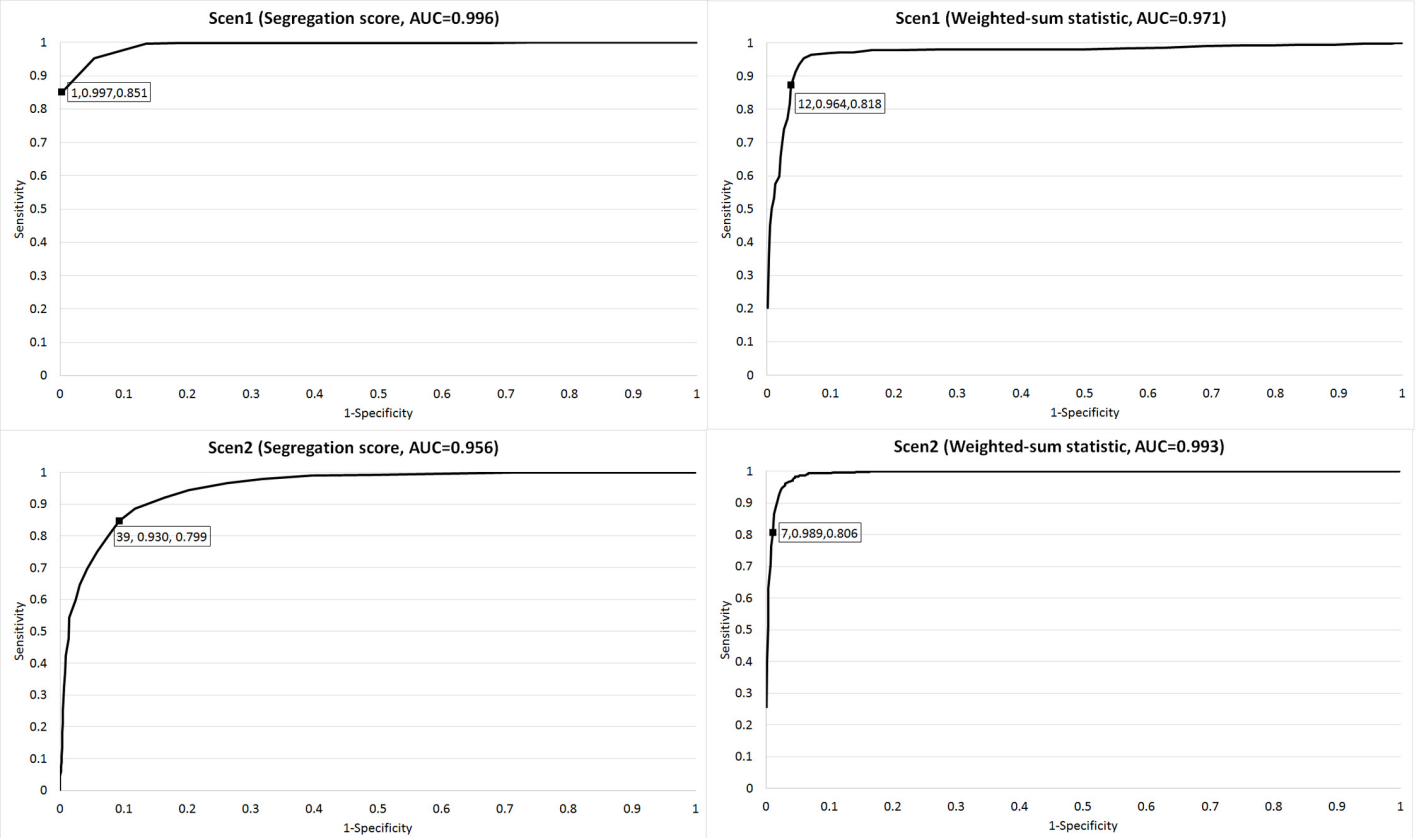
ROC curves for the Segregation score and weighted-sum statistic under Scen1 and Scen2 as described in S1 Text. A data label in the Figure shows the rank cut-off value, specificity, and sensitivity for the data point.

Fig 2 shows the receiver operating characteristic (ROC) curves for the segregation score and weighted-sum statistic under Scen1 and Scen2. Under Scen1, where family-specific mutations for the disease were simulated, the segregation score had a higher AUC (i.e., 0.996) than that (i.e., 0.971) for the weighted-sum statistic. The segregation score had a sensitivity of 85% with a 99% specificity using the rank cut-off value of 1, while the weighted-sum statistic required the rank cut-off value of 12 to achieve a similar sensitivity of 82% with a 96% specificity. Conversely, under Scen2, where different mutations in the same gene for the disease were simulated across 10 unrelated cases, the weighted-sum statistic had a higher AUC (i.e., 0.993) than that (i.e., 0.956) for the segregation score. The weighted-sum statistic had a sensitivity of 81% with a 99% specificity using the rank cut-off value of 7, while the segregation score achieved a similar sensitivity of 80% with a 93% specificity using a larger rank cut-off value of 39. Under Scen3, the filtering rules had a sensitivity of 83% with a 100% specificity, while the weighted-sum statistic required the rank cut-off value of 33 to achieve a similar sensitivity of 84% with a specificity of 83%. The simulation results demonstrated that each analysis tool in the DMI module had its advantage under a specific scenario.

**Fig 3:**
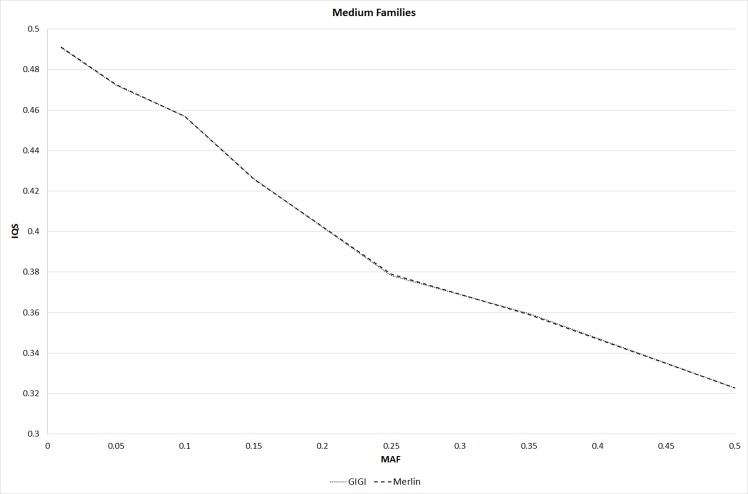
IQS for GIGI and Merlin across different intervals of MAFs for the medium families.

**Fig 4:**
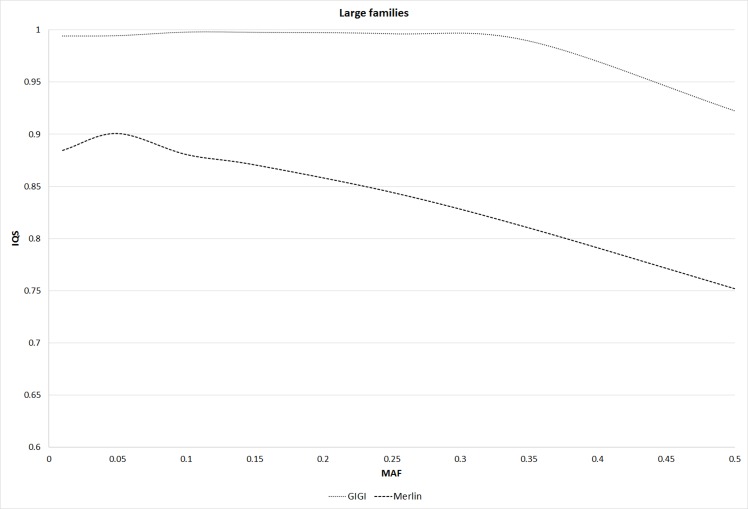
IQS for GIGI and Merlin across different intervals of MAFs for the large families.

For the imputation analysis, there were 15,505 variants in the 5 MB region, and 1,180 from the 15,505 variants were in the sparse set. Variants in four individuals from each of the three-generation families consisting of 12 individuals per family were imputed. Moreover, variants in 46 individuals from each of the large families consisting of 69 individuals per family were also imputed. Fig 3 shows the IQS for Merlin and GIGI under different MAF intervals for the medium families (i.e., the three-generation families). The IQS was similar in Merlin and GIGI across different MAF intervals, and IQS decreased with increasing MAFs for both methods. For large pedigrees, shown in Fig 4, IQS for GIGI was higher than that for Merlin. This finding is expected because GIGI used the full pedigree structure to infer the inheritance patterns, while pedigrees were split into smaller sub-pedigrees for Merlin, which resulted in loss of information for the imputations. In Merlin and GIGI, the average run times for imputing the medium pedigrees over 10 replicates of simulated pedigrees were 0.9 and 1.9 hours, respectively, whereas the average run times for imputing large pedigrees were 180.5 and 39.5 hours, respectively. Merlin spent substantially more time imputing large pedigrees because they were split into sub-pedigrees that were each imputed. Because Merlin and GIGI had similar IQS for medium pedigrees, but Merlin ran more than twice as fast as GIGI, Merlin is recommended for imputation analysis of pedigrees that are not split. However, GIGI should be used for imputing large pedigrees because it imputed with greater accuracy and efficiency than Merlin.

In conclusion, simulation results showed that for a Mendelian disorder, the segregation score is suitable to identify family-specific disease mutations when an extended pedigree is analyzed, the weighted-sum statistic is suitable for identifying disease mutations in multiple variants within a gene when multiple unrelated samples are analyzed, and the filtering rules are suitable for identifying compound heterozygosity within a gene. For pedigrees that do not need to be split, Merlin is recommended for the imputation analysis because of its efficient running time. However, for large pedigrees, GIGI should be used because it has a higher imputation accuracy than Merlin.

### Comparison of FamPipe with Other Family-Based Analysis Pipelines

Table 1 shows the comparison of FamPipe with other family-based analysis tools or pipelines. As seen in the Table, FamPipe provides more comprehensive functions than other existing tools. Although Merlin and MORGAN are also multi-functional family-based analysis tools, FamPipe presents with several advantages over the two tools. The parametric linkage functions in Merlin and MORGAN can be used to identify chromosomal regions harboring the disease variants assuming a dominant or recessive model. However, linkage analysis generally identifies a large chromosomal region, while the algorithms in the DMI module in FamPipe can be further used to identify the signal at the variant or gene level in the linkage region. Moreover, FamPipe takes advantages of the IBD output from Merlin to calculate the IBD sharing statistics. IBD and linkage regions are automatically defined by FamPipe based on the Merlin or MORGAN outputs so that imputations or association tests can be automatically performed in the regions. Furthermore, FamPipe includes GIGI, which is another useful imputation tool, for analyzing large pedigrees and two family-based statistical association tests for disease studies. Most importantly, many tedious steps to prepare the Merlin and MORGAN input files, such as the genetic positions in the map file, splitting large pedigrees for Merlin, and external allele frequencies, are all automated in FamPipe.

**Table 1:** Comparisons among different family-based analysis pipelines for sequencing data.

	Disease model	IBD analysis	Linkage analysis	Imputation	Statistical association test for disease
FamPipe	○	○	○	○	○
Merlin	○	○	○	○	
MORGAN	○	○	○		
VAR-MD	○				
FamAnn	○				
VariantDB	○				
MendelScan	○	○			
Olorin		○			
RVSharing		○			
pVAAST					○
Weighted-sum statistic					○

A **○** represents that the function is implemented in the tool.

### Availability and Future Directions

FamPipe can be freely and anonymously downloaded in source code form from http://fampipe.sourceforge.net. It is under the GNU GPL license. Currently FamPipe focuses on using SNPs for the analyses. As indels can also play an important role in disease etiology [47], one of our future aims is to incorporate indels in the analysis pipeline. In addition, one of the advantages of family-based analysis is that de novo mutations can be explicitly identified by comparing sequences between parents and offspring. Several tools have been developed to identify *de novo* mutations in families, such as DeNovoGear [48], PedigreeCaller [49], and FamSeq [50]. We hope to integrate these tools into FamPipe in the near future.

## Supporting Information

S1 Text Simulation study designs. (DOCX)
