# Supplementary material for: FamPipe: An Automatic Analysis Pipeline for Analyzing Sequencing Data in Families for Disease Studies
Source: PLoS Comput Biol. 2016 Jun 6;12(6):e1004980. doi: 10.1371/journal.pcbi.1004980 (PMC4894624; doi:10.1371/journal.pcbi.1004980)
Supplement: S1 Text — (DOCX) [file pcbi.1004980.s001.docx]

**Simulation study designs**

Simulation study for the DMI module

We compared the performance of the three methods (i.e., the segregation score, the weighted-sum p-value, and the filtering rules for compound heterozygosity) implemented in the DMI module under different scenarios. The DMI module was designed to accommodate three different analysis strategies. The first strategy is to identify family-specific mutations associated with the disease. Therefore, in the first simulation scenario (Scen1), we simulated a three-generation pedigree with 17 individuals shown in the following figure (Figure A). SeqSIMLA2_exact [1] was used to simulate the pedigree with the given affection status with the following procedures. HAPGEN2 [2] was initially used to simulate 10,000 haplotypes in a 50 MB region on chromosome 1 containing 21,849 exonic variants in 737 genes based on the 1000 Genomes Project data for the European population. Based on the 10,000 haplotypes, SeqSIMLA2_exact simulated sequences in the specified pedigree structures and affection status by calculating a retrospective likelihood. We assumed that families with a rare Mendelian disease were analyzed. Five rare variants with minor allele frequencies (MAFs) between 0.0001 and 0.001 in a gene containing 36 exonic variants were selected as the disease sites, and the penetrance function was assumed as complete dominant. Note that the weighted-sum p-value requires a population of controls. Therefore, we also used SeqSIMLA2 to simulate the same 50 MB regions in 1,000 controls. We generated genotyping errors in the simulated data based on the genotyping error rates estimated from a real dataset. More details about estimating the genotyping error rates are provided in the next section. The segregation score and the weighted-sum p-value were compared using the AUC (area under the curve). A segregation score assuming dominant model was calculated for each variant, and then the variants were sorted by the scores. The highest rank among all variants in a gene was assigned as the rank to the gene. For the weighted-sum statistic, genes were sorted by their p-values of the weighted-sum tests. A rank cut-off value was used to determine whether genes with ranks less than the cut-off value had variants following the disease model. Sensitivity was calculated based on the number of times that an algorithm correctly classified the gene containing the disease variants into the dominant model over 1,000 replicates. Similarly, the same gene simulated under the null hypothesis that no variants had effects on the disease was used to calculate the specificity over 1,000 replicates.

The second strategy of the DMI module is to identify a gene harboring several mutations at different disease causal variants in several families or unrelated affected individuals. In this simulation scenario (Scen2), SeqSIMLA2_exact was again used to simulate the 50 MB region in 10 unrelated cases and the same 5 diseases variants were selected. The segregation score and the weighted-sum p-value were also compared using the AUC. The third strategy of the DMI module is to identify a gene containing variants with compound heterozygosity. In this simulation scenario (Scen3), SeqSIMLA2_exact was used to simulate the same three-generation pedigree with 17 individuals with that in Scen1. The same 5 disease variants were assumed to have the effects of compound heterozygosity. The weighted-sum p-value and the filtering rules were compared in terms of sensitivity and specificity.


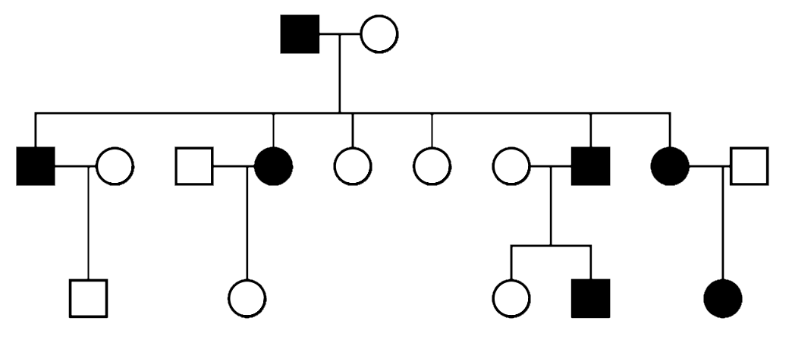


Figure A. An extended pedigree with 17 members.

Simulation study for the imputation module

We also used simulations to evaluate the performance of the two family-based imputation tools (Merlin and GIGI) included in FamPipe. A total of 10,000 haplotypes in a 5 MB region randomly selected from the 50 MB region on chromosome 1 were also simulated using HAPGEN2 based on the 1000 Genomes Project data for the European population. SeqSIMLA2_exact was used to simulate two types of family structure, shown in the following figure (Figure B), representing a medium pedigree consisting of 12 individuals and a large pedigree with 69 individuals based on the 10,000 haplotypes. We simulated 20 pedigrees for each type of pedigree structure. A sparse set of single-nucleotide polymorphisms (SNPs) with weak LD (LD measure *r^2^* < 0.5) were selected using the PLINK [3] SNP pruning function, and this set represented SNP array data. ExomePicks (http://genome.sph.umich.edu/wiki/ExomePicks) was used to select individuals in the simulated pedigrees that were sequenced. Only SNPs on the SNP array for individuals not selected by ExomePicks were retained. Variants in individuals not selected by ExomePicks were imputed, and the imputed genotypes were compared with the true genotypes. Note that the SNP array data were further pruned by FamPipe based on the procedures for generating a set of independent SNPs as described in the main text (i.e., selection of SNPs with minor allele frequencies > 0.2, pruning SNPs based on LD, and removal of SNPs with genetic distance less than 0.5 cM to the previous SNPs) for the imputation analysis. The Imputation Quality Score (IQS) [4], which accounts for the random agreement between true and imputed genotypes, was applied to assess the imputation quality of the two methods.


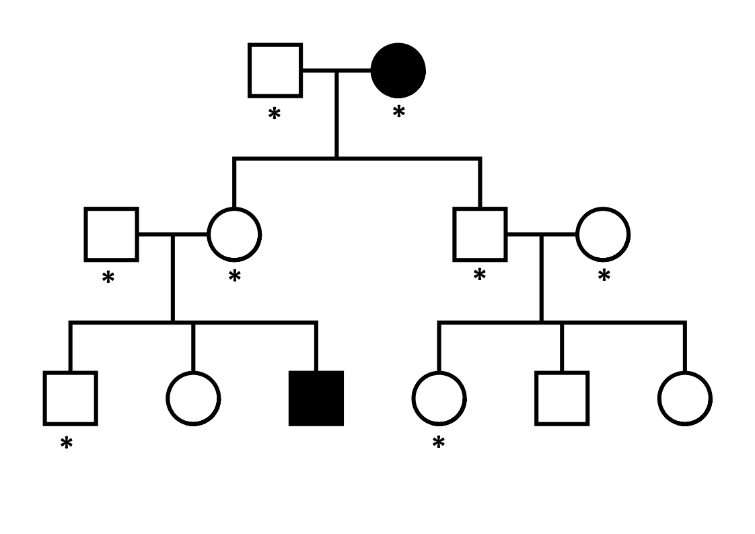


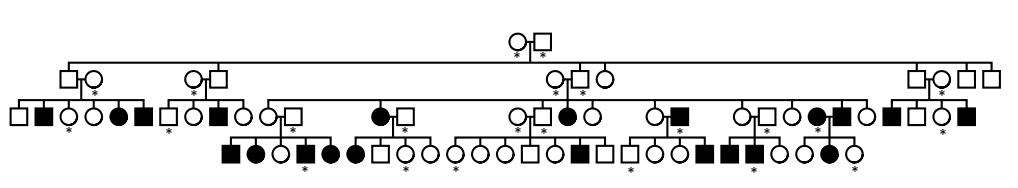


Figure B. A medium and a large pedigree structure used in the simulations for comparing the imputation accuracy between Merlin and GIGI. Individuals marked with * were selected by ExomePick.

Estimation of genotyping error rates

Two pedigrees with whole exome sequencing data were used to estimate the genotyping error rates. The pedigree structures are shown in the following figure (Figure C). Note that Family 2 in Figure B is the family used in the simulation study for the DMI module. Five individuals in each pedigree were whole-exome sequenced using Illumina HiSeq 2000 and the best practices of workflows for variant analysis in GATK [5] were performed for variant genotype calling and quality control (QC). Assuming the parental genotypes were correctly called, genotyping error rates for *DD* can be calculated based on the percentages of children whose genotypes are not *DD* but both of their parents have genotypes of *DD*. Similarly, families where both parental genotypes are *dd* and families where one parental genotype is *DD* and the other parental genotype is *dd* can be used to determine the genotyping error rates for the genotypes of *dd* and *Dd*, respectively. Note that variants with both heterozygous parents were not used in the genotyping error calculations, and we assumed that ignoring such information did not bias the estimates.


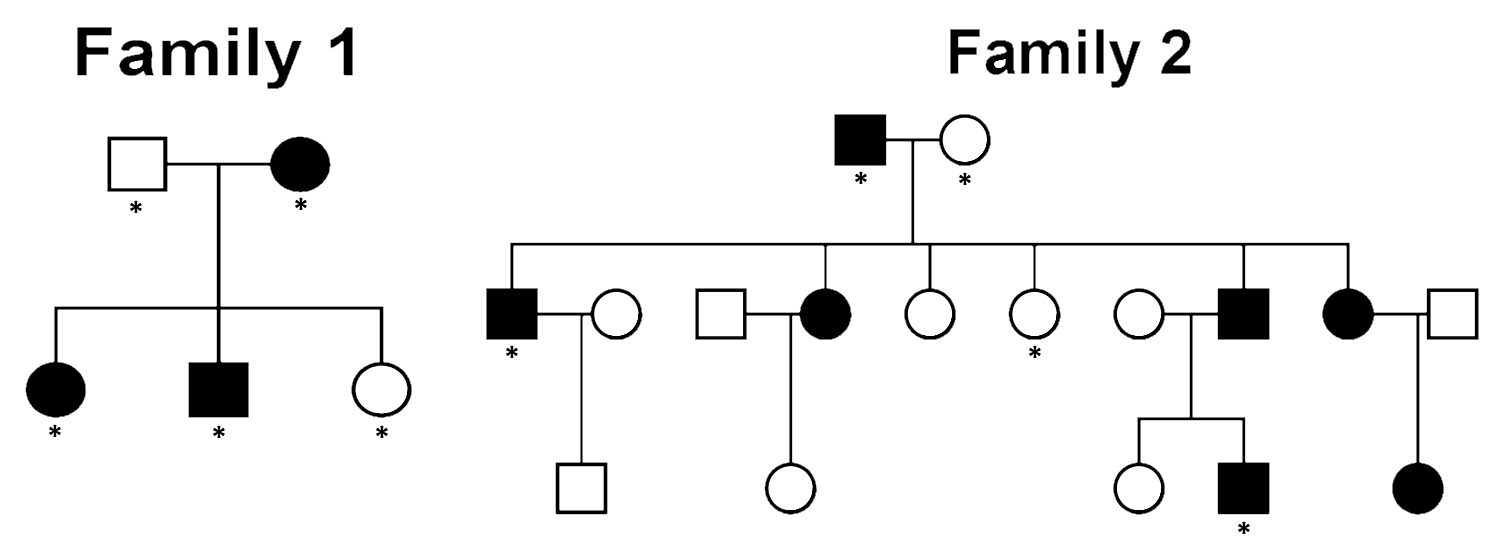


Figure C. The two pedigrees for genotyping error estimation. Individuals marked with * were sequenced.

**References**

1. Yao PJ, Chung RH (2015) SeqSIMLA2_exact: simulate multiple disease sites in large pedigrees with given disease status for diseases with low prevalence. Bioinformatics.

2. Su Z, Marchini J, Donnelly P (2011) HAPGEN2: simulation of multiple disease SNPs. Bioinformatics 27: 2304-2305.

3. Purcell S, Neale B, Todd-Brown K, Thomas L, Ferreira MA, et al. (2007) PLINK: a tool set for whole-genome association and population-based linkage analyses. Am J Hum Genet 81: 559-575.

4. Lin P, Hartz SM, Zhang Z, Saccone SF, Wang J, et al. (2010) A new statistic to evaluate imputation reliability. PLoS One 5: e9697.

5. McKenna A, Hanna M, Banks E, Sivachenko A, Cibulskis K, et al. (2010) The Genome Analysis Toolkit: a MapReduce framework for analyzing next-generation DNA sequencing data. Genome Res 20: 1297-1303.
